# Analysis of factors related to recanalization of portal vein thrombosis in liver cirrhosis: a retrospective cohort study

**DOI:** 10.1186/s12876-024-03322-8

**Published:** 2024-07-13

**Authors:** Yali Shi, Wanlin Feng, Jiaman Cai, Zhonglin Wang, Ying Pu, Weiting Mao, Ke Zhan, Daorong Chen

**Affiliations:** Department of Gastroenterology, The First Affiliated Hospital of Chongqing Medical University, 1 Youyi Road, Yuzhong District, Chongqing, 400010 China

**Keywords:** Portal vein thrombosis, Liver cirrhosis, Recanalization, Anticoagulation

## Abstract

**Background:**

Portal vein thrombosis (PVT) is a common complication of liver cirrhosis, yet there are fewer studies about predictors of PVT recanalization. We aimed to further explore the predictors of recanalization in cirrhotic PVT to facilitate accurate prediction of patients’ clinical status and timely initiation of appropriate treatment and interventions. To further investigate the benefits and risks of anticoagulant therapy in cirrhotic PVT patients.

**Methods:**

A retrospective cohort study of patients with cirrhotic PVT in our hospital between January 2016 and December 2022, The primary endpoint was to analyze predictors of PVT recanalization by COX regression. Others included bleeding rate, liver function, and mortality.

**Results:**

This study included a total of 82 patients, with 30 in the recanalization group and 52 in the non-recanalization group. Anticoagulation therapy was the only independent protective factor for portal vein thrombosis recanalization and the independent risk factors included massive ascites, history of splenectomy, Child-Pugh B/C class, and main trunk width of the portal vein. Anticoagulation therapy was associated with a significantly higher rate of PVT recanalization (75.9% vs. 20%, log-rank *P* < 0.001) and a lower rate of PVT progression (6.9% vs. 54.7%, log-rank *P* = 0.002). There was no significant difference between different anticoagulation regimens for PVT recanalization. Anticoagulation therapy did not increase the incidence of bleeding complications(*P* = 0.407). At the end of the study follow-up, Child-Pugh classification, MELD score, and albumin level were better in the anticoagulation group than in the non-anticoagulation group. There was no significant difference in 2-year survival between the two groups.

**Conclusion:**

Anticoagulation, massive ascites, history of splenectomy, Child-Pugh B/C class, and main portal vein width were associated with portal vein thrombosis recanalization. Anticoagulation may increase the rate of PVT recanalization and decrease the rate of PVT progression without increasing the rate of bleeding. Anticoagulation may be beneficial in improving liver function in patients with PVT in cirrhosis.

## Background

Portal vein thrombosis is one of the common complications in patients with cirrhosis, with a prevalence of 5-20% [1, 2]. The mechanisms of portal vein thrombosis include decreased velocity of blood flow in the portal vein, local vascular injury, and inflammation [3]. Patients with PVT in cirrhosis have an insidious onset and are usually asymptomatic, especially in the early post-onset period, so it tends to be overlooked in the diagnosis and treatment process. PVT may increase the risk of bleeding, ascites, acute kidney injury, and death after liver transplantation in patients with cirrhosis [4]. The effect of PVT on the course of cirrhosis and its overall prognostic significance is unclear. A large prospective study of the incidence of PVT in cirrhosis failed to show an association between PVT and progression of cirrhosis [5]. While others have suggested that PVT may increase the risk of long-term death in patients [4, 6]. Anticoagulation is one of the main treatments for PVT in cirrhosis, most previous reports agree with the view that anticoagulation contributes to thrombus regression and PV recanalization and that the risk of bleeding during treatment is tolerable [7–9]. And some studies have shown that the use of anticoagulation in patients with PVT in cirrhosis is correlated with improvement in liver function and survival [8, 10]; Whereas other studies do not approve [11, 12]. There is little evidence about the impact on long-term prognosis after anticoagulation, and it remains controversial whether anticoagulation improves survival in patients with PVT. Currently, the occurrence and development of PVT is unpredictable, and a few aspects of its pathophysiology, prognosis, and treatment remain unknown; Therefore, predictors of portal vein recanalization should be considered to identify patients who may not benefit from anticoagulation.

Accordingly, we aimed to further explore the predictors of recanalization in cirrhotic PVT in order to accurately predict the clinical status of patients and initiate appropriate treatment and interventions in time. To further investigate the benefits and risks of anticoagulant therapy in cirrhotic PVT patients.

## Methods

### Study cohort and data collection

**Fig. 1:**
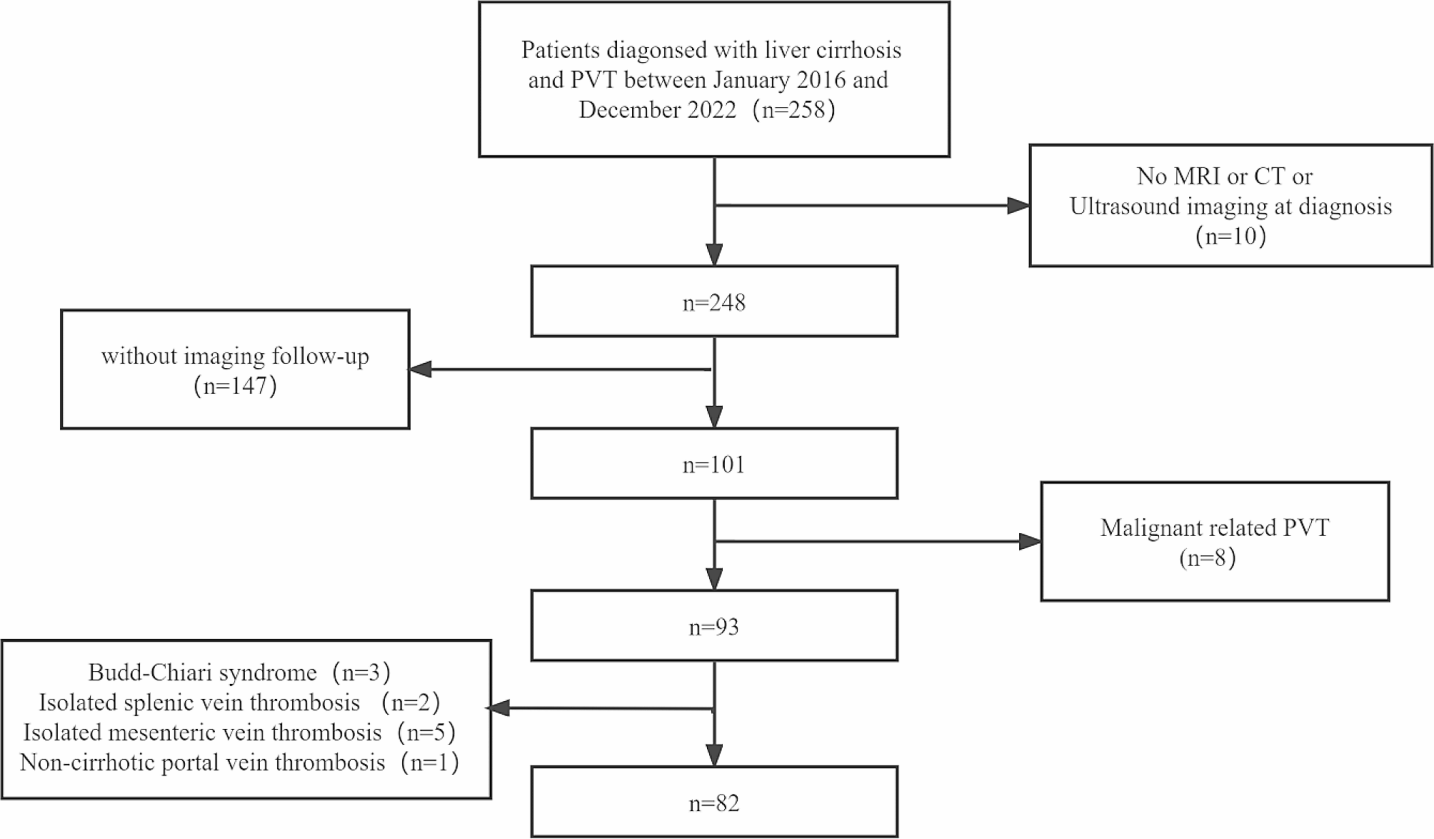
Flowchart of patients included in the study

All patients with portal vein thrombosis in liver cirrhosis at our hospital from January 2016 to December 2022 were retrospectively evaluated. The inclusion criteria were as follows: (1)liver cirrhosis was diagnosed according to the criteria of the the Japanese Society of Gastroenterology (JSGE) [13]; (2)PVT was diagnosed by abdominal Doppler ultrasound, computed tomography(CT) and magnetic resonance imaging (MRI). The exclusion criteria were: (1)Malignancy-related portal vein thrombosis; (2)Isolated mesenteric vein thrombosis or splenic vein thrombosis; (3)Budd-Chiari syndrome; (4)Primary Portal Venous Thrombosis; (5)Patients without imaging and laboratory data follow up; (6)The follow-up time less than 3 months. Figure 1 illustrates the screening flow chart. Ethical approval for this retrospective study was obtained from the First Affiliated Hospital of Chongqing Medical University.

### Follow-up and clinical end-points

Patients are followed up until death, liver transplantation, or the end of the study (May 2023). Loss to follow-up patients were tracked until their last record within our medical record system. The date of diagnosis of PVT was considered as time zero for computing follow-up. Laboratory and radiological data were collected at the time of diagnosis of PVT and at intervals of every 6 ± 3months. At the time of initial diagnosis of portal vein thrombosis, we gathered clinical data from patients through our medical record system. This data included demographic information, body mass index, comorbidities, Child-Pugh classification, MELD score, smoking and alcohol consumption history, history of ruptured esophagogastric variceal bleeding, abdominal surgery, and infection history, splenectomy history, laboratory and imaging data, anticoagulation status, and endoscopic treatment history. The primary endpoints were: PVT recanalization or progression. The location and extent of PVT occlusion and changes were assessed based on the abdominal MRI or CT report. (1) Recanalization of PVT includes complete recanalization and partial recanalization. The criteria for recanalization of portal vein thrombosis refer to the Expert Consensus on the Management of Portal Vein Thrombosis in Cirrhosis (Shanghai, 2020) [3], in which complete recanalization of a thrombus is defined as complete disappearance of the thrombus on follow-up images, and partial recanalization is defined as a reduction in the severity of the thrombus by at least one level compared with the previous thrombus, but the thrombus still exists. For example, patients with complete PVT had at least partial recanalization, or patients with partial PVT had at least 25% recanalization of the thrombus. PVT recanalization includes both complete recanalization and partial recanalization, with complete recanalization referring to complete disappearance of the thrombus and partial recanalization referring to a reduction in thrombus of more than 25%. (2) In contrast, progression of PVT is defined as an expansion of the portal vein thrombosis area or extension of the thrombus into other lumens. PVT recanalization or progression was determined only in patients who underwent imaging assessment more than 3 months after diagnosis. The median follow-up time was 9.25 months for all patients, 9.75 months for the no recanalization group, and 8.5 months for the recanalization group. The secondary outcomes were bleeding, the progress of liver function, PVT progression, and mortality.

### Statistical analysis

The SPSS software (version 26.0, IBM Corp, Armonk, NY, USA) was utilized to conduct the data analysis. Continuous variables were presented as mean ± standard deviation or median (interquartile range) and continuous variables conforming to a normal distribution were analyzed using the independent sample t-test, continuous variables that do not conform to a normal distribution were indicated by the median (interquartile range) and analyzed by nonparametric tests. While categorical data were presented as frequencies (percentages) and were compared using the χ2 test or Fischer’ s exact test. The Cox regression model was employed to perform a univariate analysis aimed at identifying potential predictors of the outcomes of PVT. Through univariate Cox regression analysis, variables with P value < 0.1 were considered confounding variables. All variables with *P* ≥ 0.1 were added one at a time in the multivariate regression model. Hazard Ratio(HR) and 95% confidence interval(CI) were calculated. Then, factors with Pvalues<0.05 were considered independent predictors after the multivariate analysis. Kaplan-Meier survival curves were used to analyze the probability of PVT recanalization and progression over time in the anticoagulation and non-anticoagulation groups.

## Result

### Baseline characteristics of included studies

258 patients with cirrhotic PVT were initially ascertained. After screening, 82 patients met the inclusion and exclusion criteria (Fig. 1). 82 patients underwent assessments to determine the progression or recanalization of PVT. PVT progressed, recanalized, and was unchanged in 31 (37.8%), 30 (36.6%), and 21 (25.6%) patients. The median time of imaging follow-up was 9.25 months (IQR-32.2 months) and did not differ significantly between the two groups (9.75 vs. 8.5 months, *P* = 0.973). 29 of these patients received anticoagulation therapy, including rivaroxaban 20 mg qd (*n* = 1), rivaroxaban 15 mg qd (*n* = 1), rivaroxaban 15 mg bid (1), rivaroxaban 10 mg qd (*n* = 4), enoxaparin sodium 4000 U qd (2), enoxaparin sodium 4000 U q12h (5), and enoxaparin sodium sequential rivaroxaban (15). Out of 29 patients who received anticoagulation, 6 had upper gastrointestinal bleeding on their hospital admission and all of them underwent endoscopic treatment before anticoagulation. Anticoagulation therapy within the following time frames after PVT detection: within 6months (*n* = 24); 7–12 months (*n* = 1); and > 12 months (*n* = 4). The median duration of anticoagulation therapy is 2 months (range, 3 days-6.5 months). Among the 29 anticoagulated patients, 2 showed progression of portal vein thrombosis (2/29, 6.9%), 22 showed recanalization (22/27, 75.9%), 12 showed gastrointestinal bleeding (12/29, 41.4%), and among the 53 patients who did not receive anticoagulation, 29 showed progression of portal vein thrombosis (29/53, 54.7%), 8 showed recanalization of PVT (8/53,15.1%), 27 showed GI bleeding (27/53, 50.9%). The baseline characteristics of the included patients are shown in Table 1.

**Table 1:** Baseline characteristics of included patients

	Total	Non-recanalization group	Recanalization group	*P*
patients(*n*)	82	52	30	
Gender				
Male	46 (56.1%)	31 (59.6%)	15 (50%)	0.398
Female	36 (43.9%)	21 (40.4%)	15 (50%)	
Age(years)	60.1 ± 12.4	59.9 ± 11.2	60.6 ± 14.4	0.783
BMI(kg/m^2^)	23.5(20.8–26.3)	23.8(20.9–26.7)	22.5 (20.8–24.7)	0.233
Hypertension	17 (20.7%)	9 (17.3%)	8 (26.7%)	0.314
Diabetes	28 (34.1%)	21 (40.4%)	7 (23.3%)	0.117
Somking	32 (39.0%)	24 (46.2%)	8 (26.7%)	0.081
Drinking	34 (41.5%)	24 (46.2%)	10 (33.3%)	0.256
Etiology				
HBV	45 (54.9%)	28 (53.8%)	17 (56.7%)	0.805
HCV	3(3.7%)	2 (3.8%)	1 (3.3%)	1.000
Alcohol	14(17.1%)	12 (23.1%)	2 (6.7%)	0.057
NASH	6(7.3%)	2 (3.8%)	4 (13.3%)	0.251
Other	14(17.1%)	8 (15.4%)	6 (20%)	0.593
MELD Score	11 (9–14)	11(9–14)	10(8–12)	0.217
Ascites				
No	16 (19.5%)	10 (19.2%)	6 (20%)	0.933
Low - medium	46 (56.1%)	30 (57.7%)	16 (53.3%)	0.702
Massive	20 (24.4%)	12 (23.1%)	8 (26.7%)	0.715
Hepatic encephalopathy	6 (7.3%)	4 (7.7%)	2 (6.7%)	1.000
Spontaneous peritonitis	20 (24.4%)	11 (21.2%)	9 (30%)	0.369
History of splenectomy	20 (24.4%)	11 (21.2%)	9 (30%)	0.369
History of Hepatic carcinoma	6(7.3%)	1(1.9%)	5(16.7%)	0.042
History of abdominal surgery	40 (48.8%)	21 (40.4%)	19 (63.3%)	0.045
Abdominal infection	24 (29.3%)	13 (25%)	11 (36.7%)	0.263
Current GIB	29 (35.3%)	21 (40.4%)	8(26.7%)	0.211
History of GIB	50(61%)	34(65.4%)	16(53.3%)	0.281
History of endoscopic operation	41(50%)	31(59.6%)	11(36.7%)	0.088
History of blood transfusion	35 (42.7%)	24 (46.2%)	11 (36.7%)	0.403
Anticoagulant therapy	29 (35.3%)	7 (13.5%)	22 (73.3%)	<0.001
NSBB	39 (47.6%)	26 (50%)	13 (43.3%)	0.506
Child-Pugh class				
A	29 (35.3%)	20(38.5%)	9(30%)	0.440
B	45 (57.3%)	29(55.8%)	18(60%)	0.709
C	6(7.3%)	3(5.8%)	3(10%)	0.788
Degree of esophageal and gastric varices				
mild	9 (11.0%)	4(7.7%)	5(16.7)	0.218
moderate	13 (15.9%)	9(17.3)	4(13.3%)	1.000
severe	40 (48.8%)	29(55.8%)	11(36.7%)	0.280
**Laboratory data**				
WBC(10^9/L)	4.5 ( 2.9–6.7)	4.4 (2.8–6.7)	4.7 (2.9–6.3)	0.661
RBC(10^9/L)	3.29 ± 0.77	3.27 ± 0.74	3.32 ± 0.83	0.794
Hb(g/L)	93.82 ± 26.25	93.0 ± 27.4	95.20 ± 24.47	0.720
PLT	75.5(53.0-146)	72.5 (49.5–137)	83.0 (62.0-160.0)	0.225
LYM#	0.66(0.41–1.03)	0.68(0.39–1.03)	0.63(0.47–1.12)	0.931
NEUT#	2.94(1.68–4.91)	2.69(1.69-5.00)	3.46(1.64–4.91)	0.814
NEUT%	73.3 (63.2–81.0)	74.4(63.9–80.7)	72.1(62.3–81.2)	0.627
AST	29.0 (22.0–41.0)	38.0(30.5–56.0)	31.0(22.0–47.0)	0.101
ALT	37.5(28.0–50.0)	31.0(26.5–43.5)	24(19.0–34.0)	0.019
ALB	32.2 ± 6.3	32.6 ± 6.3	31.5 ± 6.1	0.439
TBIL	20.9 (12.5–32.1)	23.5(15.5–33.6)	18.2(11.5–23.7)	0.093
DBIL	5.4 (2.6–8.9)	5.9 (3.3–8.9)	4.0 (2.1–9.1)	0.394
SCR	67.0(58.0–82.0)	66.0(59.5–82.0)	68.0(57.0–73)	0.889
BUN	6.0(4.8–8.8)	5.9(4.7–8.6)	6.2(5.3–9.2)	0.470
UA	295.1 ± 101.6	309.4 ± 108.3	270.5 ± 85.0	0.095
Na+	139(137–142)	139.0(137.0-142.0)	139.0(137.0-142.0)	0.768
K+	4.0(3.6–4.4)	4.0(3.7–4.5)	3.9(3.6–4.2)	0.429
PT	16.1(14.4–17.8)	16.3(14.5–17.8)	15.8(14.4–17.5)	0.586
APTT	38.4 ± 6.9	38.5 ± 7.3	38.1 ± 6.3	0.807
PTA	67.5 ± 14.8	66.5 ± 13.9	69.1 ± 16.4	0.444
INR	1.32(1.15–1.46)	1.32(1.18–1.46)	1.30(1.12–1.46)	0.563
Fib	2.15(1.64–2.76)	2.12(1.62–2.68)	2.22(1.80–2.76)	0.743
D-Dimer	3.32(1.20–7.59)	2.92(0.92–6.09)	5.65(2.02–10.92)	0.054
FDP	9.6(4.4–17.0)	8.6(3.2–15.2)	13.1(5.2–23.6)	0.065
**Imaging data**				
Main portal vein width(mm)	17.0(15.0–20.0)	18.0(15.5–20.5)	16.7(15.0–19.0)	0.218
Degree of PVT occlusion				
Non-occlusive	69(84.1%)	43(82.7%)	26(86.7%)	0.872
Occlusive	13(15.9%)	9 (17.3%)	4(13.3%)	0.872
Location of PVT				
Main portal vein thrombosis	69 (84.1%)	42(80.8%)	27(90%)	0.430
Portal branch thrombosis	54 (65.9%)	30(57.7%)	24(80%)	0.040
Splenic vein thrombosis	11 (13.4%)	5(9.6%)	6 (20%)	0.321
SMV thrombosis	32 (39.0%)	16(30.8%)	16(53.3%)	0.044
Follow-up time of imaging data(months)	9.25(6.0–16.0)	9.75(5.75-16.0)	8.5(6.0–17.0)	0.973

BMI Body mass index, HBV hepatitis B virus, HCV hepatitis C virus, NASH non-alcoholic steatohepatitis, MELD model for end stage liver disease; GIB gastrointestinal bleeding, NSBB Non-selective beta blockers, WBC White blood cell, RBC Red blood cell, HB Hemoglobin, PLT Platelet count, LYM# lymphocyte count, NEUT# neutrophil count, NEUT% neutrophilic granulocyte percentage, AST Aspartate aminotransferase, ALT Alanine aminotransferase, ALB Albumin, TBIL Total bilirubin, DBIL Direct bilirubin, SCR Serum creatinine, PT prothrombin time, APTT active partial thromboplastin time, PTA Prothrombin activity, INR international standard ratio, Fib fibrinogen, SMV Superior mesenteric vein

### Independent influences on portal vein recanalization in liver cirrhosis

The univariate predictors of PVT recanalization events and adjusted multivariate COX regression analysis results of independent influencing factors of PVT recanalization are shown in Table 2. We identified 5 predictors of PVT recanalization: massive ascites (HR = 0.313,95% CI = 0.099–0.997), history of splenectomy (HR = 0.248, 95% CI = 0.073–0.846), Child-PughB/C Class (HR = 0.261,95% CI = 0.098–0.696), main trunk width of portal vein (HR = 0.879, 95% CI = 0.774–0.999), anticoagulation therapy(HR = 6.776,95% CI = 2.514–18.262). Multifactorial regression analysis showed that massive ascites (*P* = 0.049), history of splenectomy (*P* = 0.026), Child-Pugh class B/C (*P* = 0.007), and portal vein trunk width (*P* = 0.048) were independent risk factors for portal vein thrombosis recanalization; whereas anticoagulation (*P* < 0.001) was an independent protective factor for portal vein thrombosis recanalization.

**Table 2:** Univariate and Multivariate analysis of portal vein thrombosis recanalization events

	Univariate			Multivariate		
Variable	*P*	HR	95%CI	*P*	HR	95%CI
Gender	**0.060**	2.051	0.970–4.339	0.126	2.221	0.799–6.172
Age	0.943	1.001	0.973–1.030			
BMI(kg/m^2)	0.788	0.986	0.888–1.095			
Hypertension	**0.070**	0.463	0.201–1.065	0.266	0.486	0.136–1735
Diabetes	0.169	0.551	0.236–1.289			
Smoking	**0.059**	0.440	0.187–1.032	0.444	0.631	0.194–2.051
Drinking	0.146	0.557	0.253–1.225			
Etiology(virus/non-virus)	0.762	0.893	0.430–1.856			
Meld Score	0.985	1.001	0.888–1.129			
Massive ascites	0.197	1.727	0.752–3.965	**0.049**	0.313	0.099–0.997
Hepatic encephalopathy	0.967	0.970	0.230–4.089			
Spontaneous peritonitis	**0.039**	0.430	0.193–0.960	0.164	0.420	0.124–1.423
History of splenectomy	0.785	0.896	0.407–1.971	**0.026**	0.248	0.073–0.846
History of abdominal surgery	**0.083**	0.517	0.245–1.090	0.680	0.810	0.298–2.203
Abdominal infection	**0.082**	0.512	0.241–1.088	0.365	0.643	0.248–1.671
Current GIB	0.167	0.564	0.250–1.270			
History of GIB	**0.023**	0.426	0.204–0.887	0.251	0.508	0.160–1.615
History of endoscopic operation	0.158	0.579	0.272–1.236			
History of blood transfusion	0.392	1.384	0.657–2.914			
Anticoagulant therapy	**<0.001**	5.305	2.262–12.446	**<0.001**	6.776	2.514–18.262
NSBB	0.743	0.884	0.424–1.844			
Child-Pugh Class						
A	REF			REF		
B/C	**0.067**	0.478	0.218–1.052	**0.007**	0.261	0.098–0.696
**Degree of esophageal and** **gastric varices**						
Mild	0.656					
Moderate	0.804	0.844	0.220–3.236			
Severe	0.384	0.620	0.212–1.819			
**Laboratory data**						
WBC(10^9/L)	0.580	1.026	0.937–1.124			
RBC(10^9/L)	0.931	0.978	0.592–1.616			
Hb(g/L)	0.926	1.000	0.987–1.014			
PLT	0.197	1.002	0.999–1.005			
LYM#	0.948	0.971	0.400-2.357			
NEUT#	0.552	1.031	0.933–1.140			
NEUT%	0.124	0.985	0.966–1.004			
AST	0.668	0.997	0.984–1.011			
ALT	0.987	1.000	0.989–1.011			
ALB	0.311	0.975	0.929–1.024			
TBIL	0.248	0.982	0.952–1.013			
DBIL	0.247	1.038	0.974–1.106			
CREA	0.585	0.998	0.989–1.006			
BUN	0.166	1.063	0.975–1.160			
Na+	0.374	0.899	0.782–1.033			
K+	0.920	1.035	0.530–2.022			
PT	0.604	1.043	0.890–1.222			
APTT	0.651	1.012	0.960–1.067			
PTA	0.981	0.953	0.856–1.060			
INR	0.653	1.492	0.260–8.550			
Fib	0.665	1.093	0.731–1.634			
D-Dimer*	**0.027**	1.023	1.003–1.044	0.272	1.034	0.974–1.098
FDP*	**0.065**	1.012	0.999–1.025	0.554	0.998	0.951–1.027
**Imaging data**						
**MPV(mm)**	0.130	0.925	0.836–1.023	**0.048**	0.879	0.774–0.999
**Degree of PV occlusion**						
Occlusive/Non-occlusive	0.926	0.950	0.320–2.817			
**Location of PVT**						
Main portal vein thrombosis	0.277	0.514	0.155–1.704			
Portal branch thrombosis	**0.060**	0.424	0.173–1.038	0.843	0.883	0.258–3.025
Splenic vein thrombosis	0.946	0.969	0.391–2.403			
SMV thrombosis	0.206	0.629	0.307–1.290			

BMI Body mass index, HBV hepatitis B virus, HCV hepatitis C virus, NASH non-alcoholic steatohepatitis, MELD model for end stage liver disease; GIB gastrointestinal bleeding, NSBB Non-selective beta blockers, WBC White blood cell, RBC Red blood cell, HB Hemoglobin, PLT Platelet count, LYM# lymphocyte count, NEUT# neutrophil count,NEUT% neutrophilic granulocyte percentage, AST Aspartate aminotransferase, ALT Alanine aminotransferase, ALB Albumin, TBIL Total bilirubin, DBIL Direct bilirubin,SCR Serum creatinine, PT prothrombin time, APTT active partial thromboplastin time, PTA Prothrombin activity, INR international standard ratio,Fib fibrinogen, SMV Superior mesenteric vein

### Rate of PVT recanalization

**Fig. 2:**
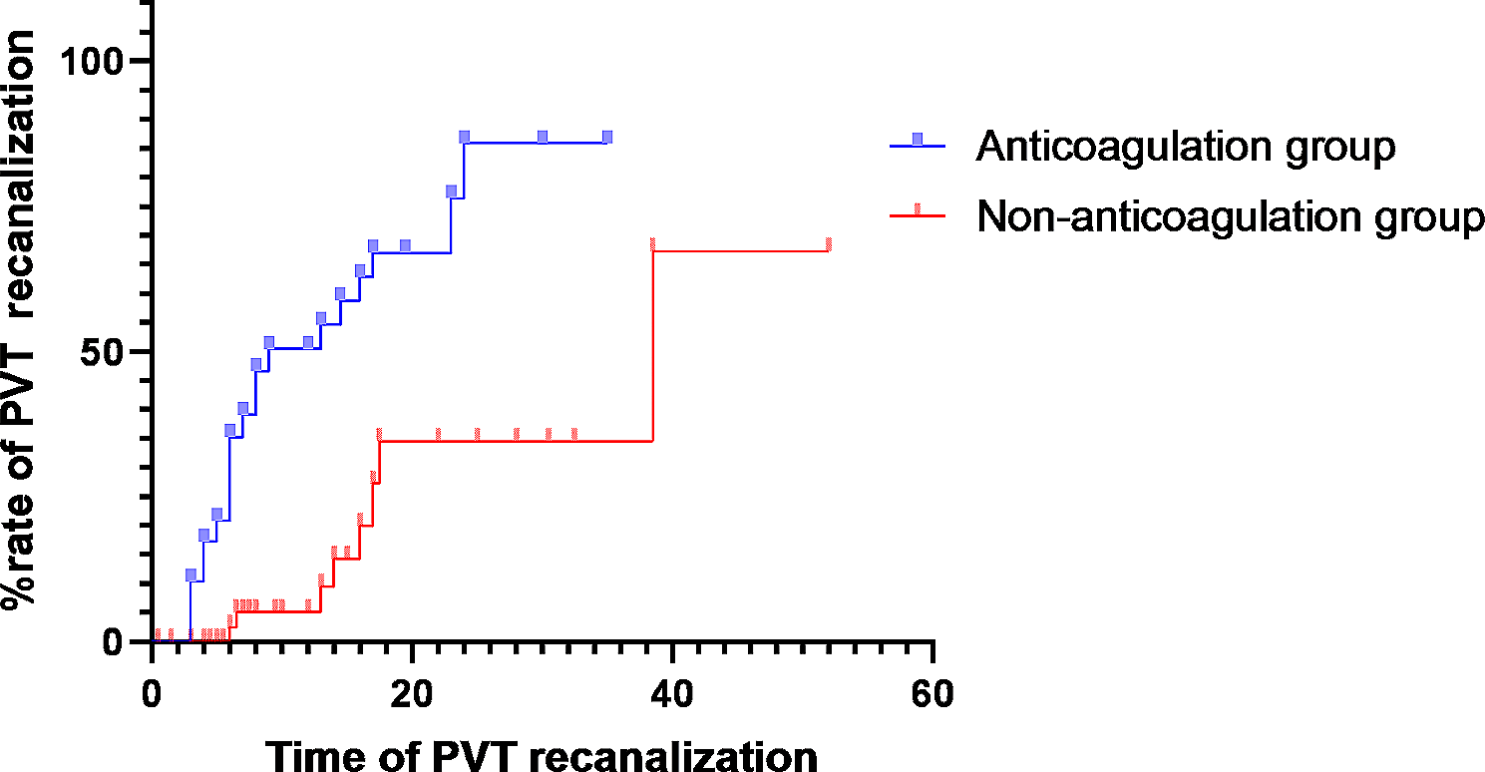
Kaplan-Meier survival curve of PVT recanalization

The Kaplan-Meier curve describes the probability of PVT recanalization in patients who received anticoagulation and those who did not, as shown in Fig. 2. PVT recanalization was observed in 22/29 (75.9) patients who received anticoagulation compared with 8/53 (15%) patients who did not receive anticoagulation (log rank, *p* ≤ 0.001). Eight patients in the anticoagulation group had complete PVT recanalization and 14 patients had partial PVT recanalization. In the non-anticoagulation group, PVT was complete recanalization in 3 patients and partial recanalization in 5 patients. Adjusted multifactorial COX regression analysis shows that anticoagulation therapy is significantly associated with increased PVT recanalization rates( HR 6.776, 95% CI 2.514–18.262, *P*<0.001). There were no significant differences in PVT recanalization rates between anticoagulation regimens. (Table 3)

**Table 3:** Cox regression analysis of PVT recanalization

Anticoagulant	HR	95%CI	log-rankP
LMWH vs. Rivaroxaban	0.787	0.251–2.465	0.647
LMWH vs. LMWH + Rivaroxaba	1.160	0.413–3.256	0.771
Rivaroxaba vs. LMWH + Rivaroxaba	1.486	0.525–4.126	0.424

**Fig. 3:**
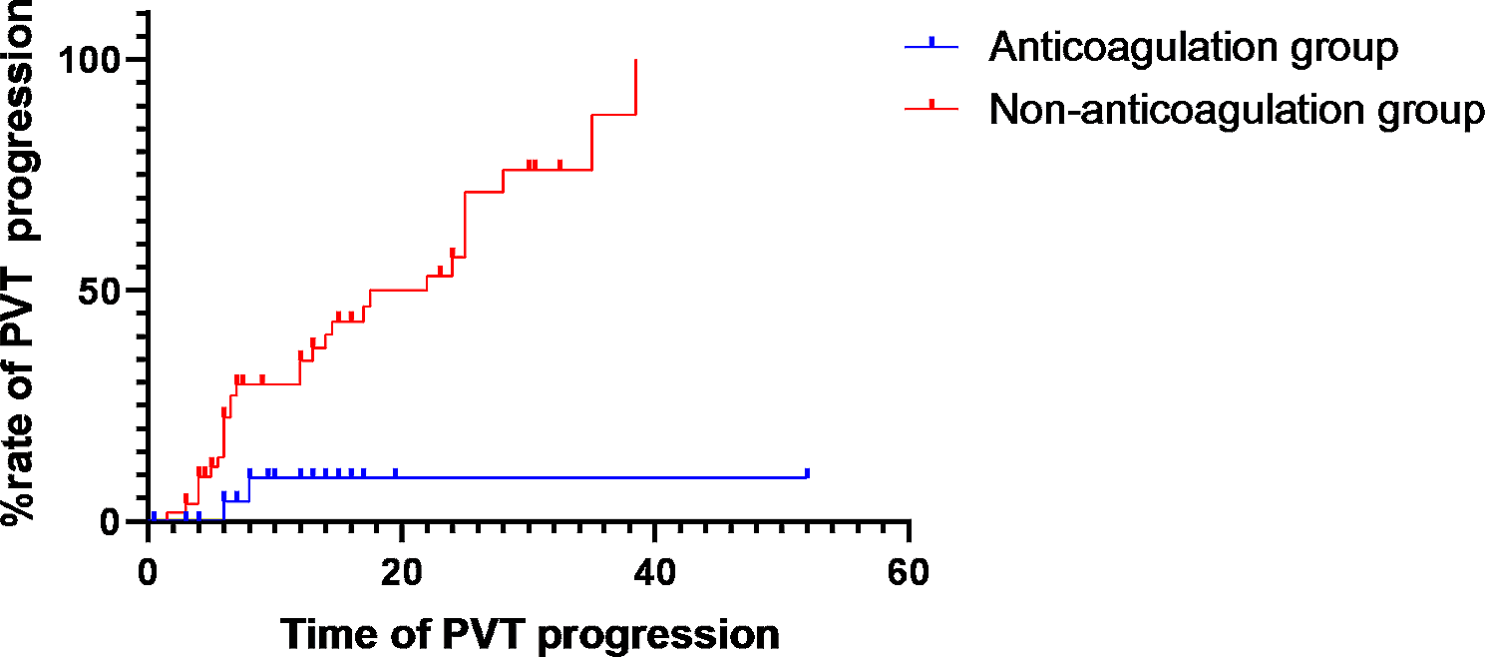
Kaplan-Meier survival curve of PVT progression

Portal vein thrombosis progression occurred in 2 of the anticoagulation patients (6.9%), compared to 29 of the non-anticoagulation patients (54.7%) (Log Rank, *P* = 0.002)(Fig. 3). Multiple cox regression analysis showed that anticoagulant application was associated with a significantly lower rate of PVT progression (HR 0.104, 95%CI 0.023–0.483, *P* = 0.004).

### Safety of anticoagulation therapy

During the follow-up period, bleeding events occurred in 12 of the 29 cases treated with anticoagulants (41.4%), including 8 cases of bleeding esophagogastric varices, 2 cases of portal hypertensive gastropathy, 1 case of peptic ulcer, 1 case of gastric fundus varicose vein degumming. Of the 53 cases without anticoagulants, 27 (50.9%) had bleeding events, including 22 cases of esophageal and gastric varices rupture and bleeding, and 5 cases of portal hypertensive gastropathy. There was no statistical significance in the incidence of bleeding complications between the two groups (*P* = 0.407). During hospitalization, anticoagulation was stopped in one patient due to low hemoglobin, in one patient due to skin ecchymosis, in three patients due to positive fecal occult blood, and in five patients anticoagulation was discontinued on its own after discharge.

### Prognostic impact of anticoagulation

We recorded serum creatinine, bilirubin, albumin, prothrombin time, International Normalized Ratio and thus calculated and compared MELD scores and Child-Pugh grades of anticoagulated versus unanticoagulated patients at the time of PVT diagnosis and follow-up. At the beginning of follow-up, there were 11 cases of Child-Pugh type A, 14 patients of Child-Pugh type B, and 3 patients of Child-Pugh type C in the anticoagulation group. There were 17 cases of Child-Pugh type A, 33 cases of Child-Pugh type B, and 3 cases of Child-Pugh type c in the non-anticoagulation group. Before follow-up, there was no statistically significant difference in liver function typing between the anticoagulation and non-anticoagulation groups (*P* = 0.436). However, at the end of the follow-up, there were 13 patients of Child-Pugh type A, 16 patients of Child-Pugh type B, and 0 patients of Child-Pugh type C in the anticoagulation group. There were 11 cases of Child-Pugh type A, 30 cases of Child-Pugh type B and 12 cases of Child-Pugh type C in the non-anticoagulation group. After follow-up, there was a statistically significant difference in liver function typing between the anticoagulation and non-anticoagulation groups (*P* = 0.006). At the end of the follow-up, the MELD score was 9 (8–14) in the anticoagulation group and 12 (9–16) in the non-anticoagulation group, with a statistically significant difference between the two groups (*p* = 0.028). Our study also found that anticoagulation therapy may help improve albumin levels, with a statistically significant difference of 35.1 ± 5.8 in the anticoagulation group compared to 32.8 ± 5.8 in the non-anticoagulation group at the end of follow-up (*p* = 0.012).

However, the difference in 2-year survival between the two groups was not statistically significant (Log Rank, *P* = 0.840). In the anticoagulation group, one case died of respiratory failure 21 months after PVT diagnosis and one case died of subarachnoid hemorrhage 24 months after diagnosis. In the non-anticoagulated group, one case died of septic shock 1.5 months after diagnosis, one case died of septic shock 3 months after diagnosis, one case died of hepatic encephalopathy 10 months after diagnosis, and one case died of respiratory failure 23 months after diagnosis. None of these deaths were related to bleeding complications.

## Discussion

PVT increases the risk of portal hypertension and related complications, such as bleeding, thrombotic progression, and death [8, 12, 14]. The presence of PVT also adds to the complexity of liver transplantation and increases the risk of early death after liver transplantation [15]. Consequently, monitoring the outcome of PVT patients’ prognosis is critical for clinicians to quickly assess PVT risk and make timely, more targeted decisions.

We analyzed the clinical outcomes of patients with PVT and identified protective and risk factors for portal vein recanalization. In our research, anticoagulation was the only independent protective factor for recanalization of portal vein thrombosis. However, the use of anticoagulants for patients with cirrhotic PVT remains controversial due to the uncertain prognosis of these patients. Additionally, anticoagulants for cirrhotic PVT have a limited suitable population recommended by the guidelines [16], and their optimal use is still unclear. This is due to insufficient clinical data on the safety and efficacy of anticoagulants for the treatment of PVT in cirrhosis. Accordingly, we analyzed the patients in our hospital to determine the current status of anticoagulants in the treatment of PVT in cirrhosis and analyzed the clinical and imaging outcomes of patients with PVT in cirrhosis to provide more experience in anticoagulation therapy. In addition, we also evaluated the impact of anticoagulation therapy on PVT. The results show that anticoagulation improves recanalization rates without increasing the risk of gastrointestinal bleeding and death rates within two years and improves liver function.

Anticoagulation is not only a common treatment but also an essential treatment option for patients with PVT and is often required in patients with non-cirrhotic portal vein thrombosis [14]. However, in patients with cirrhosis, there is controversy surrounding the need for anticoagulation. It was rarely implemented previously because clinicians and patients were concerned about complications such as gastrointestinal hemorrhage [17]. The decision of anticoagulation in the patient with cirrhosis requires consideration of the risk of hemorrhage due to portal hypertension, the severity of cirrhosis, and the potential for benefit of thrombus recanalization [18]. However, our study concluded that anticoagulation does not increase the rate of gastrointestinal bleeding, suggesting that anticoagulation is safe for patients with cirrhotic portal vein thrombosis. It could be explained by the fact that anticoagulation therapy allows recanalization of portal vein thrombus thereby reducing portal pressure, as well as the severity of esophagogastric varices, thereby reducing the incidence of gastrointestinal bleeding.The ACG Clinical Guidelines stated that anticoagulation was not associated with an increased risk of variceal bleeding in patients with hepatic cirrhosis PVT [16]. A recent randomized controlled trial by Gao et al. concluded that initiation of anticoagulation with nadroparin calcium within 48 h after EVL is safe and effective in patients with portal vein thrombosis combined with AVB [7], which provides guidance on the timing of initiation of anticoagulation for PVT combined with AVB.

Our research found that spontaneous recanalization occurred in a minority of PVT patients(15.1%), similar to the results of previous studies [15, 19], but the recanalization rate was higher in patients treated with anticoagulants, which implies that anticoagulation seems to be effective in the treatment of PVT in cirrhosis. In our study, 75.9% of patients who received anticoagulation achieved thrombus recanalization; this result was in accordance with the previous studies (30-80%) [14, 18] and also confirmed that anticoagulation is a significant predictor of PVT recanalization. Therefore, we recommend that the majority of patients with PVT should receive anticoagulation unless there is a high risk of bleeding.

In our study, the presence of massive ascites, history of splenectomy, Child-Pugh class B/C, and increased MPV width were independent risk factors for portal vein thrombosis recanalization. The appearance of ascites is one of the most characteristic manifestations of liver cirrhosis entering the decompensated phase, and massive ascites indicates liver failure in cirrhotic patients. There is a mutually reinforcing relationship between massive ascites and portal hypertension. Ascites further increases the pressure in the portal system, making blood flow more sluggish or stagnant, and this altered blood flow state provides the conditions for thrombosis. A multicenter retrospective study found that in liver cirrhosis patients with ascites as a single decompensatory event, recalcitrant ascites, spontaneous peritonitis, hepatorenal syndrome, and hepatic encephalopathy may subsequently occur, and that the incidence of further decompensatory events and the mortality rate correlate with the severity of ascites [20]. Studies have shown that ascites is also associated with PVT. Maruyama et al [12]. demonstrated that ascites is associated with the development of PVT in liver cirrhosis patients with viral hepatitis, and the percentage of patients with combined PVT who developed ascites was significantly higher than the percentage of patients without combined PVT in this study (50.00% vs. 25.93%,*p* < 0.05). In clinical practice, the width of the portal vein trunk is one of the simple indicators of increased portal vein trunk pressure. The diameter of the portal vein trunk correlates with its portal pressure. When thrombosis occurs, blood flow is obstructed, portal pressure increases, and due to compensatory widening of the portal vein trunk, some damage or compression of the vessel wall is caused, which can damage endothelial cells, increase the risk of thrombosis, and decrease the probability of recanalization of portal vein thrombosis. Decreased blood flow velocity is a contributing factor to venous thrombosis. Several studies have found by Doppler ultrasonography that the risk of portal vein thrombosis in cirrhotic patients increases 10–20 times if their portal vein blood flow velocity is less than 15 cm/Sects. [21, 22]. In portal hypertension, the body reduces the pressure in order to compensate for the widening of the portal vein trunk, and the wider the inner diameter of the portal vein trunk, the higher the portal vein pressure, the slower the portal vein blood flow velocity, the increase in blood platelet and coagulation factor adhesion and aggregation in the blood vessel wall, and the disruption of physiological systems regulating the microcirculation of coagulation, which promotes the formation of portal vein thrombosis [21]. Portal vein thrombosis will further aggravate portal hypertension, making blood flow even slower and forming a vicious circle, thus reducing the probability of recanalization of portal vein thrombosis. Portal blood flow has been reported to be significantly lower in Child-Pugh class B and C patients than in Child-Pugh class A patients [23]. A correlation between portal blood flow and serum bilirubin or albumin levels in patients with cirrhosis was reported previously [24] which suggests that portal blood flow is at least partially dependent on liver function; therefore, the rate of portal vein recanalization over time may be lower in patients with poorer liver function (Child-Pugh classes B and C) than in patients with better liver function (Child-Pugh class A). The increase in leukocytes and blood platelets after splenectomy may cause portal thrombosis, according to several studies [25]. A majority of studies have concluded that after splenectomy, the splenic vein becomes a blind end, causing portal vein resistance, the decreased blood flow rate, and prolonged contact between coagulation factors and blood vessel walls. The destruction and reduction of platelets after splenectomy leads to a dramatic increase in platelets. At the same time, the surgery itself disrupts the vascular endothelium, which together promotes the formation of thrombosis [26], associated with lower chances of complete recanalization and longer duration of PVT.

Although most previous research agreed that anticoagulation contributes to thrombus regression and PVT recanalization, however, there is limited evidence on the long-term prognosis after anticoagulation, and whether anticoagulation improves hepatic function and survival in patients with PVT remains controversial [16]. The MELD score is a scoring system used to assess the severity of the condition of patients with end-stage liver disease, the higher the MELD score, the more severe the condition of the patient and the worse the prognosis; the Child-Pugh classification is another method of assessing liver function and prognosis of patients with cirrhosis, the lower the Child-Pugh classification, the better the liver function of the patient, and the higher the quality of life of the patient. Albumin is an important protein synthesized by the liver, which plays a key role in maintaining plasma colloid osmotic pressure, transporting nutrients, and participating in detoxification. In the case of cirrhosis, the liver’s synthetic function is impaired, leading to a decrease in albumin synthesis, which may result in hypoproteinemia. This further may lead to the development of symptoms such as ascites, pleural fluid, and lower extremity edema. Elevated albumin levels in patients with liver cirrhosis may indicate that the patient’s liver synthetic function has improved. Therefore, improvement in MELD score and Child-Pugh classification and increase in albumin level in liver cirrhosis patients after anticoagulation therapy are important indicators for assessing the effectiveness of treatment. In our study, the difference in baseline Child-Pugh classification of liver function between the anticoagulation and non-anticoagulation groups at the beginning of follow-up was not statistically significant, but at the end of follow-up, there was a statistically significant increase in Child-Pugh class C patients and a decrease in class A patients in the non-anticoagulation group. Similarly, there was no statistically significant difference in albumin levels between the anticoagulation and non-anticoagulation groups at the beginning of the follow-up, whereas albumin levels were higher in the anticoagulation group than in the non-anticoagulation group at the end of the follow-up, and the difference was statistically significant. A randomized controlled trial showed Child-Pugh scores improved after anticoagulation compared to before anticoagulation (7 vs. 6, *p* = 0.007) and the albumin level increased after anticoagulation(36.06 6 5.13 vs. 38.64 6 3.75, *p* = 0.004) [9]. Previous studies have also mentioned that anticoagulation may improve liver function [9, 27, 28], which is supported further by our study. A multicenter, long-term follow-up study of PVT in cirrhosis showed no statistically significant difference in Kaplan-Meier overall survival curves between the anticoagulation and non-anticoagulation groups after 5 years of follow-up (83% vs. 70%, log-rank *P* = 0.1362) [14]. A meta-analysis showed no statistically significant difference in 1-, 3-, and 5-year survival rates between the anticoagulation and non-anticoagulation groups [29]. The difference in 2-year survival between the two groups was not statistically significant (log-rank, *P* = 0.840)in our study. A recent meta-analysis [30] showed that anticoagulation reduced all-cause mortality in patients with cirrhosis combined with PVT, with a hazard ratio of 0.59 (95% CI 0.49–0.70). The survival benefit of anticoagulation is independent of recanalization and may be the result of reduced macrovascular and microvascular thrombosis, the latter of which is caused by endothelial dysfunction in cirrhotic hepatic sinusoids and is associated with hepatic stellate cell activation and fibrosis progression [29, 30].

Clinicians often choose not to anticoagulate or to reduce the dose of anticoagulants because they are concerned about the occurrence of bleeding. During our study, however, there was no statistically significant difference in the risk of bleeding complications during the follow-up period between patients who were anticoagulated and those who were not(*p* = 0.407), despite the fact that one patient who used anticoagulation during hospitalization had a drop in hemoglobin, one had skin ecchymosis, and three had a positive fecal occult blood that led to the discontinuation of anticoagulation. A recent meta-analysis [30] concluded that anticoagulation does not increase the risk of portal hypertension bleeding in patients with decompensated cirrhosis, whereas the incidence of non-portal hypertension-related bleeding (mainly of gastrointestinal origin) was higher in the anticoagulated group. An RCT by Gao et al [7]. demonstrated that it was safe to start nadroparin calcium–warfarin sequential anticoagulation therapy 48 h after EVL in patients with PVT combined with AVB, with four-week (2.3% vs. 4.7%, *P* = 1.000), six-week (4.7% vs. 9.3%, *P* = 0.672), and six-month hemorrhage rates (18.6% vs. 20.9%, *P* = 0.787) which were similar in both groups, demonstrating that NWS anticoagulation was safe for PVT patients with cirrhosis and AVB. However, this study included patients with better liver function reserve and did not adequately evaluate the safety of anticoagulation in cirrhotic patients with a Child-Pugh score of C. In our study, one Child-Pugh class C patient showed better safety and efficacy with LMWH-Rivaroxaban sequential therapy for 3 months, while two Child-Pugh class C patients were suspended by their physicians after 4 days and 1 month of anticoagulant therapy because of positive fecal occult blood, respectively. The safety and efficacy of anticoagulation in patients with progressive cirrhosis, especially Child-Pugh class C, deserve further evaluation. More research in the future needs to include Child-Pugh class C patients to provide more convincing evidence.

Some limitations of our study need to be taken into account. Firstly, this study was a retrospective cohort study with a limited number of patients, which may have biased the data analysis. Secondly, we did not follow patients who achieved recanalization for a longer period of time to assess whether they experienced thrombus recurrence, as well as dynamically follow patients with laboratory and imaging data and the occurrence or absence of complications to assess whether there was a difference between thrombus recanalized and nonrecanalized patients. Finally, due to the limited number of patients included, future prospective multicenter randomized clinical trials with larger sample sizes are needed to confirm the results of this study.

## Conclusion

In conclusion, we identified independent influences on portal vein recanalization in cirrhosis, which may help clinicians to identify early and initiate appropriate treatment and interventions in a timely fashion. Anticoagulation can improve PVT recanalization rates without increasing bleeding rates in cirrhotic patients, and anticoagulation may be able to improve the level of liver function in cirrhotic patients; Future studies should further optimize the regimen of anticoagulation for PVT in cirrhosis, as well as the safety of anticoagulation in cirrhotic patients with Child-Pugh class C liver disease.

## Data Availability

The datasets used and/or analyzed during the current study are available from. the corresponding author on reasonable request.

## Abbreviations

PVT: Portal vein thrombosis
GIB: Gastrointestinal bleeding
SMV: Superior mesenteric vein
CT: Computed tomography
MRI: Magnetic resonance imaging
C-index: Concordance index
HR: Hazard ratio
CI: Confidence interval
BMI: Body mass index
HBV: Hepatitis B virus
HCV: Hepatitis C virus
NASH: Non-alcoholic steatohepatitis
MELD: Model for end stage liver disease
WBC: White blood cell
RBC: Red blood cell
HB: Hemoglobin
PLT: Platelet count
LYM#: Lymphocyte count
NEUT#: Neutrophil count
NEUT%: Neutrophilic granulocyte percentage
AST: Aspartate aminotransferase
ALT: Alanine aminotransferase
ALB: Albumin
TBIL: Total bilirubin
DBIL: Direct bilirubin
SCR: Serum creatinine
PT: Prothrombin time
APTT: Active partial thromboplastin time
PTA: Prothrombin activity
INR: International standard ratio
Fib: Fibrinogen
MPV: Main portal vein width
